# Big-Data-Mining-Based Improved K-Means Algorithm for Energy Use Analysis of Coal-Fired Power Plant Units: A Case Study

**DOI:** 10.3390/e20090702

**Published:** 2018-09-13

**Authors:** Binghan Liu, Zhongguang Fu, Pengkai Wang, Lu Liu, Manda Gao, Ji Liu

**Affiliations:** 1 School of Energy, Power and Mechanical Engineering, North China Electric Power University, Beijing 102206, China; 2 National Engineering Laboratory for Biomass Power Generation Equipment, North China Electric Power University, Beijing 102206, China

**Keywords:** big data mining, coal-fired units, operation optimization, energy use analysis, K-means, sensitivity analysis

## Abstract

The energy use analysis of coal-fired power plant units is of significance for energy conservation and consumption reduction. One of the most serious problems attributed to Chinese coal-fired power plants is coal waste. Several units in one plant may experience a practical rated output situation at the same time, which may increase the coal consumption of the power plant. Here, we propose a new hybrid methodology for plant-level load optimization to minimize coal consumption for coal-fired power plants. The proposed methodology includes two parts. One part determines the reference value of the controllable operating parameters of net coal consumption under typical load conditions, based on an improved K-means algorithm and the Hadoop platform. The other part utilizes a support vector machine to determine the sensitivity coefficients of various operating parameters for the net coal consumption under different load conditions. Additionally, the fuzzy rough set attribute reduction method was employed to obtain the minimalist properties reduction method parameters to reduce the complexity of the dataset. This work is based on continuously-measured information system data from a 600 MW coal-fired power plant in China. The results show that the proposed strategy achieves high energy conservation performance. Taking the 600 MW load optimization value as an example, the optimized power supply coal consumption is 307.95 g/(kW·h) compared to the actual operating value of 313.45 g/(kW·h). It is important for coal-fired power plants to reduce their coal consumption.

## 1. Introduction

Non-renewable energy and coal comprise the majority of the resources utilized in Chinese energy use and production. As the Chinese energy structure features an abundance of coal and a shortage of oil and gas, this resource distribution is not likely to change in the near term [1]. By the end of 2015, the average coal consumption of the thermal power units, which generate 600 MW of power, was 315 (g/kW·h) [2]. According to the Chinese development plan, by 2020, coal-based power generation should account for over 60% of the total coal consumption. At the same time, coal-fired units must be upgraded to reduce emissions and conserve energy. In addition, the average coal consumption of all the active coal-fired units should be lower than 310 (g/kW·h) [3]. Owing to the rapid development of clean energy in China, thermal power units are always under a low load [4]. Determining how to attain minimum coal consumption during low-load operation is a significant challenge for those who are involved in energy production.

With the application of the supervisor information system (SIS) and distributed control system (DCS), massive amounts of power plant data can be saved. With the rapid development of data-mining technology in the power industry, many scholars have begun to use this data to optimize the operation of coal-fired power plant units [5,6]. The K-means algorithm [7] is a classical clustering algorithm. It is widely used in the optimization of power unit operations because of its simplicity and fast convergence. In Reference [8], a method was used to measure the target value of boiler monitoring parameters by realizing synchronous clustering of several parameters based on the fuzzy C-means clustering algorithm. In Reference [9], the K-means algorithm was used to measure the reference value of pressure loss in a reheater and the temperature of the boiler exhaust gas by analyzing real-time operating data. However, a large number of candidate sets and redundant datasets emerge, which reduce clustering efficiency and accuracy. Therefore, an attribute reduction method based on fuzzy rough sets is introduced in this paper. Using this method, the attributes of power units are reduced before the use of the clustering K-means algorithm, to enhance the efficiency and accuracy of the algorithm. This is achieved by eliminating redundant attributes and narrowing the data scale. The theory of fuzzy rough sets is a mathematical tool used for solving problems related to uncertainty and vagueness. By considering the operating parameters that affect the power supply coal consumption rate as an information system, this theory can be used to analyze the system and calculate the degree of dependence of certain parameters on the consumption rate by reducing their attributes. Then, redundant factors are eliminated based on their degree of dependence. As a result, the simplest influential parameter set is calculated. In addition, by relying on the initial number of clusters, the K-means algorithm might lead to a local optimal solution. In this paper, the Canopy algorithm is used. There is no need to set up the number of clusters in this algorithm. It first conducts a clustering analysis of the data to affirm the initial cluster center and the number of clusters. Next, it conducts iterative computations using the K-means algorithm. Lastly, the clustering result is achieved and the disadvantages of the K-means algorithm are avoided.

Cloud computing [10] meets the demands of massive data mining, and parallel computing is currently the most effective method [11]. To address massive and high-dimensional data, this paper introduces the MapReduce Program Mode to the parallel clustering of K-means. First, the method executes a pre-treatment of controllable operating parameters based on the fuzzy and rough sets theory. Secondly, the K-means algorithm is improved by the Canopy algorithm, and a new parallel clustering algorithm called FMK-means is realized. This algorithm is used to mine the reference value of controllable operating parameters that affect power supply coal consumption under optimal operational circumstances. Lastly, the method analyzes the sensitivity of each parameter to coal consumption under different working loads based on vector technology. This step provides guidance for the optimization and debugging of power units. Compared to traditional data mining, the new algorithm for massive data mining enhances the accuracy of clustering, eliminates redundant data sets, and promotes clustering efficiency.

## 2. Analysis of Energy Loss of Thermal Generators

Because the implementation of policies aimed toward reducing the coal consumption rate of coal-fired units has intensified, the Chinese power supply coal consumption rate has decreased each year. However, maintaining consumption reduction, safety, reliance, and environmental factors must also be taken into consideration. Therefore, small and out-of-date units should be eliminated and environmentally friendly units featuring low consumption and large capacity should be built. Heat and power cogeneration remains the dominant trend in the development of thermal generation. However, active generation units should be upgraded and optimized, and operation modes and parameters should be confirmed to ensure that units attain the best operation states under different loads. In addition, environmental pollution caused by coal can be classified as pollution due to coal burning and pollution due to coal mining. Pollution by solid particulate waste produced through coal mining is the main cause of environmental pollution. Combustible coal processing wastes are the most promising components for coal-water slurries with and without petrochemicals (CWS and CWSP). The use of coal-water slurries not only reduces the cost of coal milling, but also increases the burning efficiency of coal and reduces pollution [12,13].

**Figure 1:**
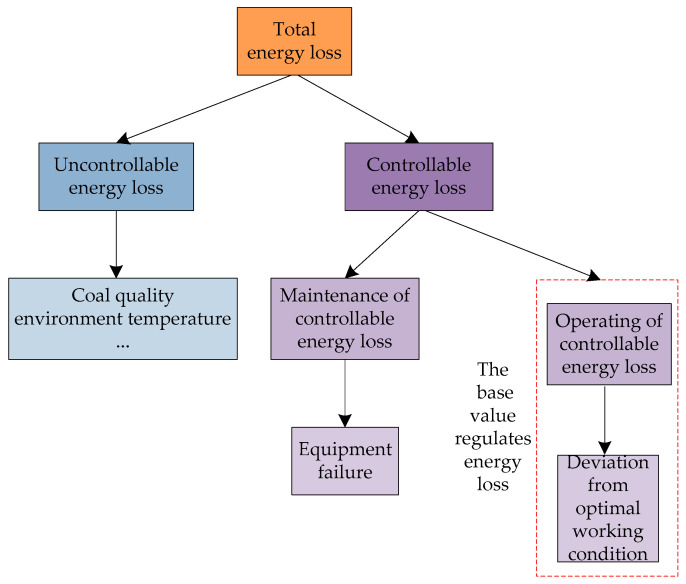
Energy use analysis of plant units.

The successful operation of an economical and energy-efficient power plant is dependent on the efficient analysis of the energy use in generator units. Generally, power plants take the power supply coal consumption rate as a standard for assessing the energy conservation and consumption level of units. Currently, energy use analysis methods often refer to the first law of thermodynamics, which is based on mass balance and energy balance, and the second law of thermodynamics, which is based on energy analysis [14]. According to the second law, maximum energy loss is caused by energy loss due to irreversible declining energy quality. Determining how to minimize energy loss caused by irreversibility is an important task for people working in the energy field. From the control perspective, the energy loss of thermal units can be classified into controllable and uncontrollable losses [15]. As shown in Figure 1, uncontrollable energy loss is caused by external factors that are difficult to improve, such as coal quality and ambient temperature. Currently, as a result of coal diversity, many power plants choose to conduct coal blending [16] and raw coal separation processes [17] before burning, to reduce the energy loss caused by changes in coal quality. Controllable energy loss can be divided into operational loss and maintenance loss. The former is caused by the deviance of units from optimal operating conditions, which is caused by the deviance of operating parameters from a reference value. At the same time, thermal performance and operating efficiency are affected. This part of the loss can be recovered by adjusting the controllable operating parameters. Maintenance controllable energy loss is usually caused by equipment failure, but this element of loss can be recovered by performing routine maintenance.

During unit operation, the equipment is relatively stable, so maintenance controllable energy loss accounts for a smaller proportion of the total loss. However, operational controllable energy loss increases significantly if the operators lack professional skills and/or do not properly supervise the units. Creating a reasonable reference value for controllable operational parameters effectively reduces operational controllable energy loss. The term “reference value” represents the reference value for each operational parameter corresponding to ideal operating circumstances. Many scholars have conducted research on the reference values of generation units in power plants [18,19]. Therefore, an accurate and reasonable reference value for operational parameters is of great significance for improving unit performance and reducing energy use.

## 3. Relevant Theories of New Algorithm

### 3.1. Fuzzy and Rough Sets Theory

In 1990, Dubois became the first to combine fuzzy sets and rough sets and proposed the fuzzy and rough sets model [20]. The fuzzy and rough sets model “softens” data sets and reduces their attributes by taking advantage of similarly formed data, based on vagueness and roughness. The upper and lower approximation sets of the fuzzy rough set are defined as follows [21]:

(1) $$u_{\underline{P}X}(F_{i})=\underset{x}{\inf}\max\left\{1-u_{F_{i}(x)},u_{X}(x)\right\},\forall i$$

(2) $$u_{\bar{P}X}(F_{i})=\underset{x}{\sup}\min\left\{u_{F_{i}(x)},u_{X}(x)\right\},\forall i$$

where *U* is a nonempty domain and *F_i_* is decision equivalence class, $F_{i}\in U/P=\left\{F_{1},F_{2},...,F_{n}\right\}$.

The fuzzy positive domain of the fuzzy and rough sets model is:

(3) $$u_{pos_{p}(Q)}(x)=\underset{X\in U/P}{\sup}u_{\underline{P}X}(x)$$

where *P* is the conditional attribute and *Q* is the decision attribute. The formula reflects the capability of the conditional attribute to characterize the decision attribute. According to the relevant definition of the fuzzy positive domain, the attribute dependence rate of the fuzzy and rough sets model can be defined as follows:

(4) $$\gamma_{P}(Q)=\frac{\left|u_{POS_{P}(Q)}(x)\right|}{U}=\frac{\sum_{x\in U}u_{POS_{P}(Q)}(x)}{U}$$

Equation (4) represents the dependence rate of decision attribute *Q* on conditional attribute *P*. It is apparent that the bigger the $\gamma_{P}(Q)$, the stronger the dependence of the decision attribute on the conditional attribute, and the closer the sample to the decision attribute.

The objective of attribute reduction is to search for the simplest characterization set of the conditional attribute to the decision attribute, and then to delete the redundant conditional attribute. The QuickReduct attribute reduction algorithm is a classical method used to accomplish this. It has been utilized in many applications because of its fast search speed and simplicity. Its operating principle is to select an empty set R, and then add those attributes that increase dependence rate $\gamma_{R}(D)$ to set R until $\gamma_{R}(D)$ reaches its maximum value. A flow diagram of the attribute reduction algorithm is presented as follows:

**Algorithm 1.** The QuickReduct attribute reduction algorithm.1: $R\leftarrow \left\{\right\},\gamma_{best}\leftarrow 0,\gamma_{prev}\leftarrow 0$ 2: Do 3: T←R 4: $\gamma_{prev}\leftarrow \gamma_{best}$ 5: ∀x∈{C−R} 6: $if\;\gamma_{R\cup \left\{X\right\}}(D)>\gamma_{T}(D)$ 7:   T←R∪{x} 8:   $\gamma_{best}\leftarrow \gamma_{T}(D)$ 9: R←T 10: $until\;\gamma_{best}=\gamma_{prev}$ 11: return R

### 3.2. Canopy Algorithm

The algorithmic thought of Canopy [22] means that for massive data, a Canopy refers to an algorithm that divides the input data points into several overlapping clusters by using the distance measuring method, and then the points in the Canopy are clustered by a computing method with high accuracy.

**Definition** **1.** *Canopy: There is a given dataset $F=\{f_{i}|i=1,2,\cdots ,n\}$, if $\forall x_{i}\in F$, and ${\{c}_{j},|\exists ||x_{i}-c_{j}||\leq D_{1},c_{j}\subseteq F,i\neq j\}$; then, the set of $x_{i}$ is called the Canopy set, $c_{j}$ is the center point of the Canopy, and $D_{1}$ is the semi-diameter of Canopy set.*

**Definition** **2.** *The center point of Canopy: there is a given dataset $F=\{f_{i}|i=1,2,\cdots ,n\}$, if $\forall x_{i}\in F$, and ${\{c}_{n},|\exists ||x_{i}-c_{n}||\leq D_{2},D_{2}<D_{1},c_{n}\subseteq F,i\neq n\}$; then, c_n_ is the set of the non-candidate center points of the Canopy.*

### 3.3. K-Means Clustering

K-means is defined as a classic unsupervised learning algorithm that is based on the partition clustering method. Its basic algorithmic process is described as follows. After randomly selecting *k* data points in the raw data set, the initial values of these points are taken as the center of each cluster. Then, the distance between non-central data points and the center of each cluster is calculated, and the data points are allocated to the cluster nearest to them. After these points are clustered, the mean of each cluster is calculated and the center point of each cluster is selected once again. This process is repeated until the objective criterion function converges [23]. The definition of the criterion function is defined as follows:

(5) $$E=\sum_{i=1}^{k}\sum_{x\in C_{i}}{\left(x-\bar{x_{i}}\right)}^{2}$$

where *E* is the sum of the squared Euclidean distances between each data point and its corresponding cluster center, *x* is a point in the data space, and $\bar{x_{i}}$ is the arithmetic mean of each cluster. By adopting the criterion function, the generated clusters can be as compact as possible, whereas the different clusters can be as independent as possible.

To count the number of clusters calculated by K-means, this paper introduces the RSS (Ressidual Sum of Squares) variance function, which can vectorize the data in K-means. In addition, the selected *k* data points can be regarded as the center of the vectors; the computational formula is defined as follows:

(6) $${\vec{x}}_{i}=\frac{1}{\left|C_{i}\right|}\sum_{\vec{x}\in C_{i}}\vec{x}$$

(7) $$RSS_{k}={\sum_{\vec{x}\in C_{i}}\left|\vec{x}-{\vec{x}}_{i}\right|}^{2}$$

(8) $$RSS=\sum_{k=1}^{k}RSS_{k}$$

where *RSS_k_* is the distance between each data point in class *k* and the center, and *RSS* is the sum of the *RSS_k_* in all *k* of classes.

### 3.4. Hadoop Platform

Owing to its high throughput and efficiency, good reliability, and automatic fault tolerance, the Hadoop platform has been widely used to address massive data in recent years. HDFS (Hadoop Distributed File System) and MapReduce comprise the core design of this platform; the former can store massive amounts of data, whereas the latter can compute the large collections of data.

HDFS, the data storage management framework of Hadoop, has high reliability. It can copy all data blocks and store them in three independent slave nodes. Thus, if one of the data blocks is lost, copies of the data block are available to be called.

**Figure 2:**
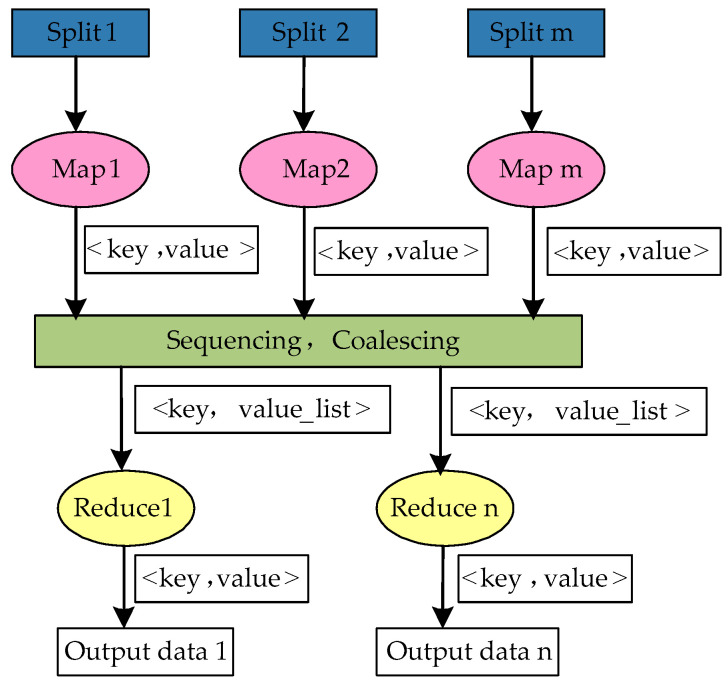
MapReduce execution flowchart.

As the data computing framework of Hadoop, MapReduce is utilized to execute parallel processing of the distributed models of massive data. Map and Reduce are the two major functions of this framework. Data blocks are input and output in the form of <key, value>. The output values of data blocks are calculated by the independent and parallel Map function, then these output values are sequenced and merged. Finally, the operation results whose key values are equal are taken as the input value of the Reduce function, which are reduced and output. The computing process of MapReduce is shown in Figure 2 [24].

## 4. Calculation Process of FMK-Means Algorithm

**Figure 3:**
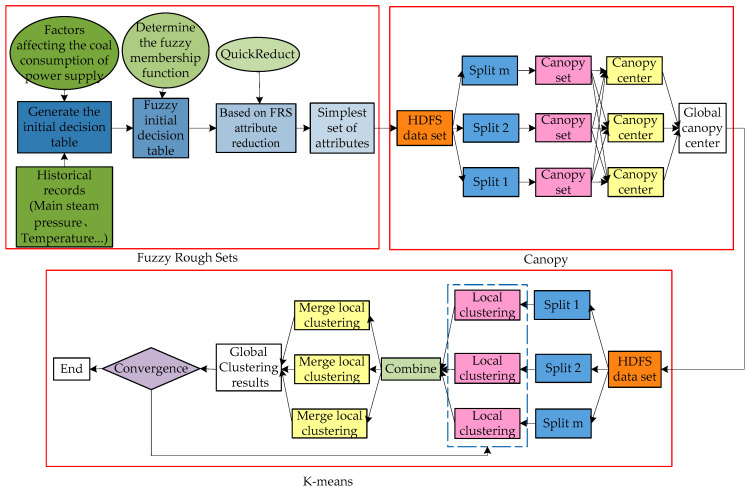
Flowchart of FMK-means algorithm execution.

This paper adopts the FMK (Improvement of K-means algorithm by the fuzzy rough set and MapReduce)-means algorithm to obtain the reference value of the controllable operating parameter under optimal conditions, which is closely related to the power supply coal consumption rate. Firstly, the algorithm reduces the attributes of each operating parameter using the fuzzy and rough sets model. After the candidate attributes have been reduced, the calculated simplest set of attributes is converted into the programming model for MapReduce, thus realizing the parallel processing of the improved K-means algorithm. Then, the center point of the optimal cluster for each operating parameter is identified. The algorithm flow of FMK-means is presented in Figure 3, and the procedure is detailed below:

(1) Establish a decision table of energy use. Consider the various factors that may affect power supply coal consumption and divide the operating conditions according to external conditions, such as load, coal type, and environment temperature. Take the coal consumption rate as the decision attribute, and the controllable operating parameters, which have a close relation with the former, as the condition attribute.
(2) Clarify the fuzzy membership function in accordance with the attributes of each parameter and convert the parameter data into set y of fuzzy data.
(3) Reduce the operating parameters that affect the coal consumption rate by using the QuickReduct method. After the simplest set of attributes has been calculated, compute the importance of each condition attribute to the coal consumption rate.
(4) In the Map stage of Canopy, convert the simplest set of attributes into the form of <key, value> and send these key values to m in Map functions. Then, calculate the distance threshold of each data point and compare these distances with D1 and D2. After being classified, these distances are iterated into the Canopy set.
(5) In the Reduce stage of Canopy, unite and calculate the output of the Map stage and form the dataset Q; then, process the set Q using Canopy. Repeat the above steps until the dataset becomes empty, and then obtain the cluster K and its center point, which is taken as the input value of the K-means framework.
(6) In the Map stage of K-means, convert the reduced new data set into the form of <key, value> and send it to *m* in Map functions. Calculate the distance between each node data and each cluster center, and then allocate these nodes to the cluster that is nearest to them. Mark each cluster type and output them in the form of <key, value>.
(7) The Combine function is used to divide the output value of the Map function, then merges the data that belong to the same cluster. Sum the corresponding dimension of the data in one cluster and count the number of data objects. Finally, the calculated results are output in the form of <key, value>. “Key” is the type of cluster, whereas “value” is the corresponding dimension of data and the accumulated number of the data objects.
(8) In the Reduce stage of K-means, receive the output value of the Combine function, then analyze the sum of the corresponding dimension of the data in each cluster, as well as the total number of data objects. Thus, new cluster centers will be obtained, and a new round of iteration will be conducted until the function converges.

## 5. Energy Use Sensitivity Analysis Using Support Vector Machine (SVM)

The energy use sensitivity of a machine refers to the variation in its energy use, which is caused by the operating parameter deviating from the reference value or the designed value [25]. In actual operation, it is helpful to reduce the power supply coal consumption rate by identifying the influence that the operating parameters of the machine exert on the coal consumption rate. This is achieved by paying attention to the relatively high sensitivity coefficient. Based on the SVM, this paper has established a function that can describe the relation between the power supply coal consumption and controllable operating parameters. By calculating the partial derivative of the coal consumption rate to each parameter, and then acquiring the response characteristics, the sensitivity of each operating parameter to coal consumption can be analyzed and confirmed. Therefore, this paper can provide reference for unit optimization and commissioning in practice.

Establish a sample set $\vec{D}=\{({\vec{x}}_{1},z_{1}),({\vec{x}}_{2},z_{2}),\ldots ,({\vec{x}}_{i},z_{i}),\ldots ,({\vec{x}}_{n},z_{n})\}$ for power supply coal consumption and operating parameters. ${\vec{x}}_{i}={\left\{x_{i1},x_{i2},\ldots ,x_{ij},\ldots ,x_{im}\right\}}^{T}$ is the influence vector of the *i*-th sample, i=1,2,…,n, *n* is the number of samples, j=1,2,…,m, and *m* is the number of operating parameters. Then set up a mathematical description function of the power supply coal consumption rate and each operating parameter:

(9) $$z=f(x)=f(x_{1},x_{2},\ldots ,x_{i},\ldots ,x_{n})$$

In this paper, the nonlinear low space is mapped into the high space and a linear regression model is created, thus establishing a mathematical model for coal consumption and operating parameters:

(10) $$z=f(x)=\sum_{i=1}^{n}(a_{i}-a_{i}^{*})k(x_{i},x)+b$$

where $a_{i}$ and $a_{i}^{*}$ are the Lagrange multipliers, and $k(x_{i},x)$ is a kernel function.

Calculate the sensitivity coefficient of the coal consumption rate by using the linear kernel function $k(x_{i},x)=x_{i}^{T}x$ [26]:

(11) $$f(x)=\sum_{i=1}^{n}\sum_{j=1}^{m}(a_{i}-a_{i}^{*})x_{ij}x_{j}+b$$

Calculate the partial derivative of Equation (8) and obtain an influencing parameter $x_{j}$ of the coal consumption rate:

(12) $$\frac{\partial_{f(x)}}{\partial_{x}}=\sum_{i=1}^{n}(a_{i}-a_{i}^{*})x_{ij}$$

The value of $\partial_{f(x)}/\partial_{x}$ directly indicates the sensitivity coefficient of coal consumption rate to parameter $x_{j}$, based on which, a sensitivity analysis model of coal consumption of power supply can be established:

(13) $$\Delta z=\sum_{j=1}^{m}\left[\sum_{i=1}^{n}(a_{i}-a_{i}^{*})x_{ij}\right]\Delta x_{j}$$

## 6. Example Analysis

### 6.1. Object and Goals of Study

Based on the accumulated massive data in the Distributed Control System (DCS) and the rigorous arithmetic logic, big data mining technology can extract the factors affecting the coal consumption rate by analyzing the operational data of the thermodynamic system. Although the cumulative reference value of the optimal operating parameter has certain discrepancies with the theoretical value, this technology shows the reachable optimal values of the operating parameters among all the records. We selected a unit from a 600 MW coal-fired power plant for further study. The boiler is a primary reheat subcritical controlled circulation drum boiler (HG-2023/17.6-YM4), and the turbine is a subcritical reverse condensing turbine (N600-16.7/537/537-1). A total of 129,600 samples of operating data from March 2013 to May 2013 were adopted, and the sampling period was 60 s.

There are many different factors that affect the coal consumption rate in a thermal unit. In addition to the oxygen content in exhaust gas, main steam temperature, reheat steam temperature, and other internal factors, external factors, such as the quality of the coal and ambient temperature, are equally important. Because it is difficult to analyze the nature of coal in real time at present, the net calorific value of fired coal was chosen to replace the former in this paper. The range of the net calorific value of coal ranged from 21.35 to 22.15 MJ/Kg, and the ambient temperature was within 20 to 26 °C. Under certain external conditions, we adopted the actual controllable operating parameters that were closely related to the coal consumption rate. With the aid of the new algorithms derived from the big data of FMK-means, as well as the corresponding relationship between the cluster centers of operating parameter and the coal consumption rate, the reference value of the controllable operating parameter under optimal conditions was calculated. The selected operating parameters are listed in Table 1.

The following section analyzes the influence of partially controllable operating parameters on the power supply coal consumption rate and the relationship between them. In addition, the relative parameters were selected based on the above analyses.

(1) Main steam pressure: When the main steam temperature, exhaust pressure, and reheat stream parameter remain unchanged and the main steam pressure is lowered, the ideal enthalpy drop of the unit decreases and the turbine steam rate increases, which are accompanied by a drop in unit power. Thus, the efficiency and security of the unit inevitably degrades. It can be concluded that the deviation of main steam pressure from the reference value affects the efficiency and security of the unit.
(2) Main stream temperature: When the parameters of main stream pressure, exhaust pressure, and reheat steam are constant, and the main steam temperature is reduced, the ideal enthalpy drop of the unit and its efficiency decrease and exhaust humidity increase, resulting in a decrease in unit power. Thus, the economy of the unit decreases. It can be seen that the changes in main stream temperature influence the coal consumption rate.
(3) Reheat stream temperature: Similar to the main stream temperature analysis, the changes in reheat stream temperature influence unit economy and safety. When the reheat stream temperature deviates from the reference value, the work capacity loss and coal consumption rate increase.
(4) Emission capacity of oxygen content: The emission capacity of oxygen content is the excess air coefficient. If the excess air coefficient is too small, it increases the incomplete combustion loss of the unit and reduces combustion capacity, thereby reducing unit efficiency. If the coefficient is too large, it cannot only reduce the incomplete combustion loss and unit efficiency; it will increase the exhaust smoke loss. Therefore, choosing an appropriate range for the excess air coefficient is crucial for improving the efficiency and economic operation of the unit.
(5) Feedwater temperature: The change in feedwater temperature also leads to economic changes in the unit. Reducing the feedwater temperature increases the heat absorption of the working substance in the water-cooled wall and lower the exhaust temperature to a certain extent. However, the amount of fuel must be increased to maintain a certain amount of evaporation, which leads to an increase in furnace outlet temperature and each part of gas temperature. As a result of the two effects, the economy of the unit decreases. Therefore, it is necessary to maintain the appropriate feedwater temperature.

### 6.2. Algorithm Application and Calculation Result

The new FMK-means algorithm was used for large data mining. The power supply coal consumption rate was taken as the decision attribute and the other operating parameters were used as the condition attributes. In order to avoid the influence of individual bad data and noisy data, the dependency threshold value was set as Δγ=0.05; when the attribute dependency increment was greater than Δγ, other attributes were stipulated in the reduction. The dependency increments of the reheat steam pressure, the primary reheat desuperheating water temperature, the secondary reheat desuperheating water temperature, as well as the pressure and temperature of condensate water, were 0.0056, 0.0072, 0.0064, 0.0012, and 0.0015, respectively. All of the dependency increases were less than Δγ, so they were considered to be unnecessary attributes. The final attributes were reduced to RED (Reduction) (P) = {A1, A2, A3, A5, A6, A7, A8}; the dependency of each parameter was calculated from definition formulation given in Section 3.1, which is shown in Table 2.

**Figure 4:**
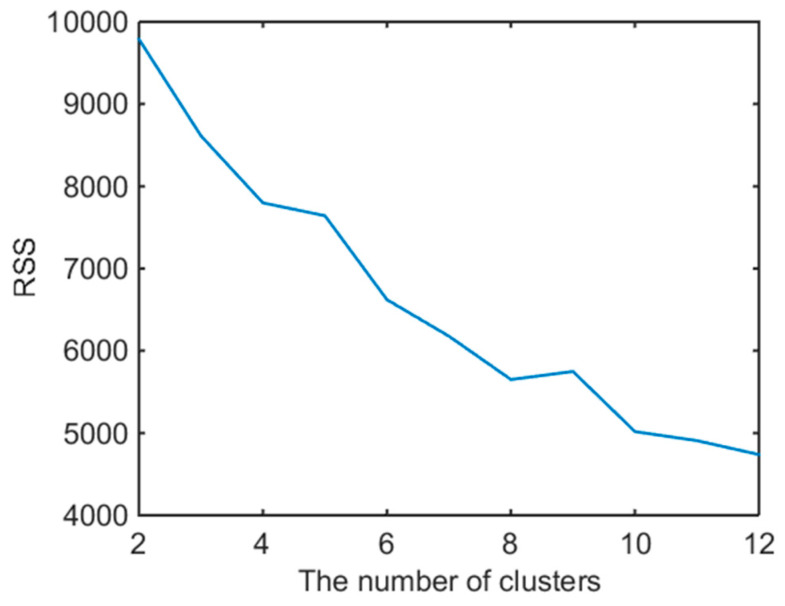
Clustering number and RSS curve.

**Figure 5:**
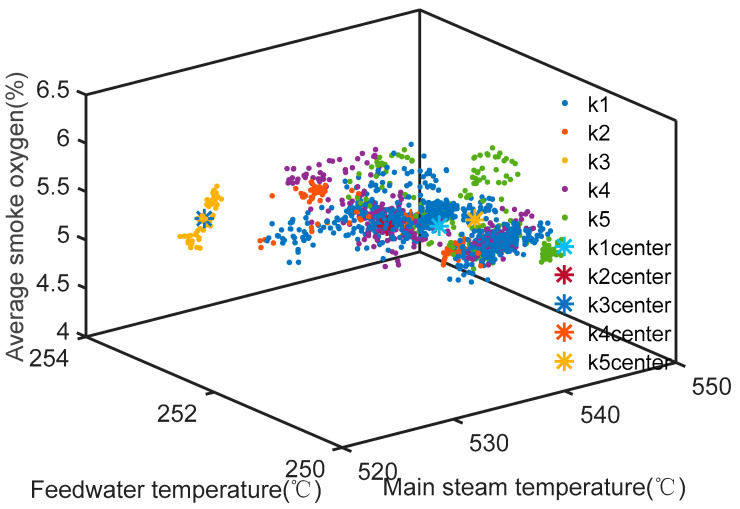
Clustering of the main steam temperature water supply temperature and exhaust oxygen at the condition of 400 MW.

The reduced attribute index was applied to the MapReduce programming model for K-means parallel cluster processing, and the Hadoop platform was set with a minimum support of 2%; that is, the number of data clustering under a load was not less than 2% of the total number. Based on the clustering results under a 400 MW load condition, the RSS variance function was used to obtain the clustering *k* function graph of RSS, as shown in Figure 4. When the RSS dropped from a significant decline to a *k* value whose decrease was not as obvious, it could be used as the final clustering number *k*. Then, the classification number, *k* = 5, was optimal for the clustering results, which are shown in Table 3. The coal consumption rate was the lowest in the second categories, and there were enough data tuples to locate a sample point. The clustering results of the main steam temperature, feedwater temperature, and emission capacity of oxygen content are shown in Figure 5. The sampling points of the clustering results under different load conditions were determined, and the minimum values of coal consumption and corresponding controllable operation parameters were excavated. The results are listed in Table 4.

The reference values of the controllable operating parameters that affect the power supply coal consumption rate, which was calculated by the new FMK-means algorithm, and the optimal target value of the unit can be seen in Table 4. (1) Under load conditions of 300, 400, 500, and 600 MW, the actual values of the unit coal consumption rate were 320.69, 312.76, 310.90, and 307.95 g/(kW·h), respectively; (2) Taking the 600 MW load optimization value as an example, the optimized value of power supply coal consumption was 307.95 g/(kW·h) compared to the actual operating value of 313.45 g/(kW·h). This indicates a reduction of 5.5 g/(kW·h). If the optimization method is adopted in the operation of a unit under the 600 MW load condition, the unit can save 5.5 g of coal per 1 kW·h of power generation. While saving the amount of coal for power generation, it not only reduces the coal consumption and pollutant emissions, but also serves as an energy conservation mechanism.

### 6.3. Analysis of Energy Use Sensitivity Under Different Loads

**Figure 6:**
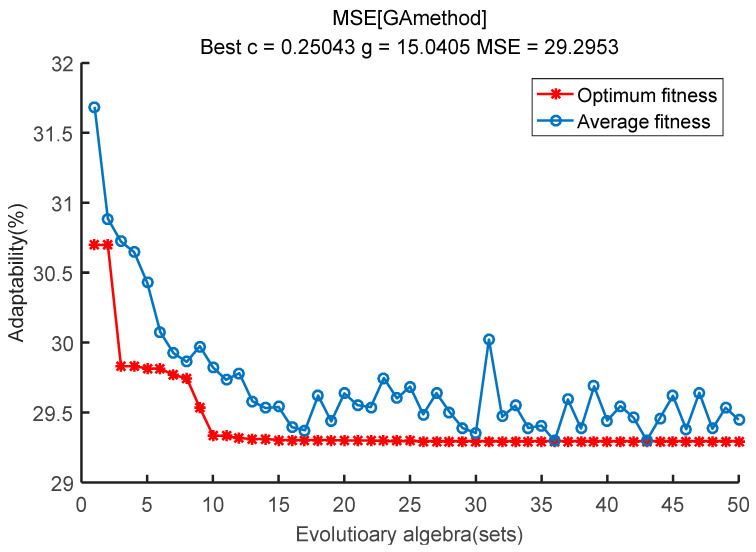
Genetic algorithm parameter optimization.

**Figure 7:**
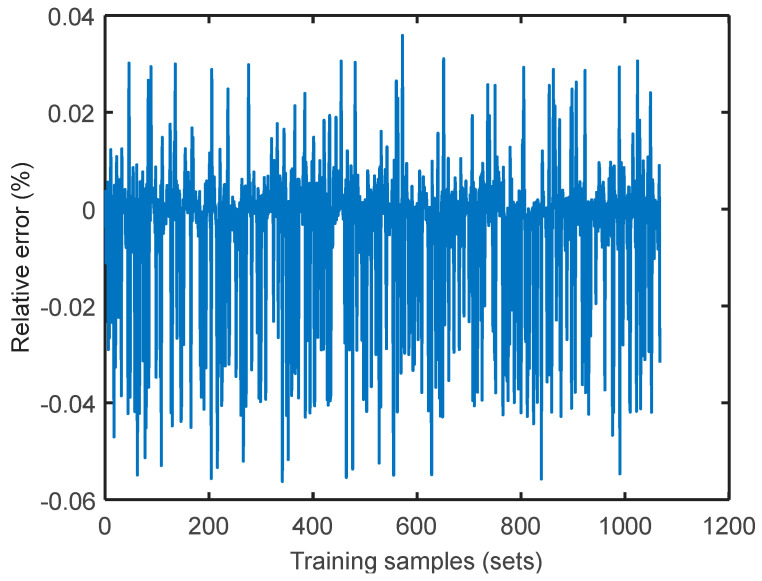
Relative error curve of training samples.

**Figure 8:**
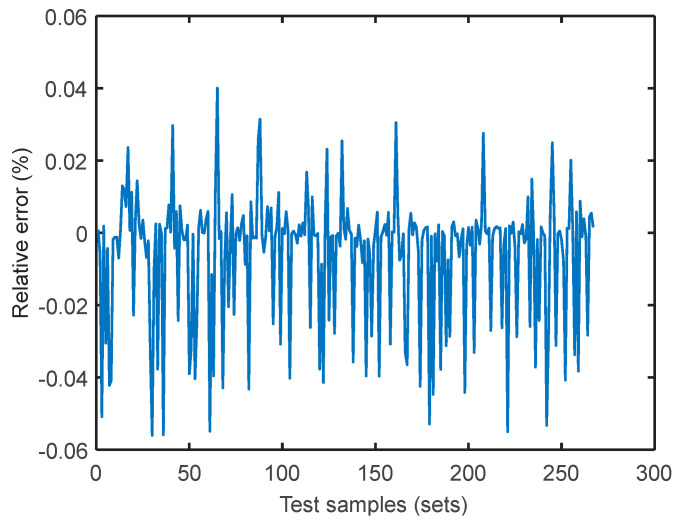
Relative error curve of testing samples.

Take the simplest attribute parameter of the fuzzy and rough attribute reduction as the input model and the coal consumption rate as the modeling target to calculate the sensitivity coefficient under the load conditions of 300, 350, 400, 450, 500, 550, and 600 MW using the SVM coal consumption sensitivity analysis model built in Section 4. Taking the sensitivity coefficient under the 400 MW load condition as an example, apply the Genetic Algorithm for data reprocessing and the calculation of parameter iteration optimization to select the optimum parameters *c* and *g* (the better the parameters *c* and *g*, the better the SVM fit) as the input parameters of SVM. The results are listed in Figure 6. The relative error curves of the training and testing samples under the 400 MW load condition are shown in Figure 7 and Figure 8, respectively, in which the sample accuracy is basically within ±0.04% (less than 0.5%). Therefore, it was practicable to calculate the sensitivity coefficient of the operation parameter for coal consumption with the SVM algorithm. The sensitivity coefficient value of each operation parameter under the 400 MW load condition was calculated by the SVM energy use analysis model, which is shown in Table 5. As such, the sensitivity coefficients of each operation parameter under different load conditions can be calculated.

The sensitivity analysis model of power supply coal consumption based on SVM was used to calculate the sensitivity coefficient of each parameter for coal consumption under different load conditions. The number of training data sets, test data sets, and SVMs, as well as the average error of training data and test data, are provided in Table 6. The sensitivity coefficient calculation results of each parameter for the coal consumption under the load conditions of 300, 350, 400, 450, 500, 550, and 600 MW are listed in Table 7.

From the above calculation results, we drew the following determinations:

(1) The number of samples in each load interval was larger than that of the modeling support vectors (Table 6). Instead of using all the samples, the typical sample training model that can be used as a support vector is applied to modeling, which can not only reduce the complexity of training samples but also the time spent in modeling.
(2) The model accuracy of the 300, 350, and 400 MW load condition was lower than that of the 450, 500, 550, and 600 MW load condition (Table 6). The reason is that the higher the load, the more stable the units, which results in the reduction in noise data in the high load area and decreased influence on modeling. Thus, the model accuracy in the high load area is higher.
(3) The operating parameters under different load sensitivity coefficients on the power supply coal consumption was constantly changing (Table 7), which indicates that the influence of all operating parameters on coal consumption is different if the load condition is not the same. In addition, the relationship between the sensitivity coefficient and the load was non-linear; therefore, it is necessary to analyze the sensitivity coefficient of each parameter for the coal consumption under typical load conditions. In actual unit operating processes, corresponding measures should be adopted to reduce the coal consumption in accordance with different load conditions, and measures should be taken first for the parameters with high sensitivity coefficients.

## 7. Discussion

In recent years, many researchers have utilized data mining techniques to develop methods for coal saving in coal-fired power plants. A previous study [4] reported the combination of different data mining methods to construct a plant-level load optimization model. When the model was used for the simulation of three different types of power plants, the maximum reduction of coal consumption for power generation in coal-fired power plants could be achieved. In the present study, big data mining methods were used to calculate the baseline values and sensitivity coefficients of controllable operating parameters affecting coal consumption for power generation in a stand-alone coal-fired power plant. The operating data of the thermodynamic system were subsequently analyzed to investigate the effectiveness of the proposed methods on the reduction of coal consumption. The clustering results in Figure 5 and the optimization results in Table 4 clearly demonstrate that the methods used in the present study effectively lower coal consumption for power generation and substantially reduce coal usage. The support vector machine-based method for the calculation of sensitivity of controllable operating parameters developed in this study can also provide guidance for the reduction of coal consumption during actual operations of power plants.

## 8. Conclusions

To conduct energy use analysis and examine the energy efficiency of coal-fired power stations, this paper takes controllable operating parameters as the starting points and adopts a large amount of operating data to study algorithm and SVM technology. The objective was to determine controllable operating parameter reference values and sensitivity coefficients that influence the degree of coal consumption within a thermal unit. The results of the study are as follows:

(1) The introduction of the Fuzzy and Rough Sets Theory and the Canopy algorithm improved the K-means clustering algorithm. The improved K-means algorithm was then subjected to parallel processing by the MapReduce programming model to study the new FMK-means algorithm, which eliminated redundant data and greatly improved clustering accuracy and efficiency.
(2) The multi-index data mining of the historical data of a 600 MW coal-fired generating units was conducted by using the new FMK-means algorithm. The algorithm was able to determine the controllable operating parameter reference values and the actual reachable values of coal consumption under the optimal working conditions and provides guidance regarding how to adjust the operation of the unit. Under load conditions of 300, 400, 500, and 600 MW, the actual values of the unit coal consumption rate were 320.69, 312.76, 310.90, and 307.95 g/(kW·h), respectively.
(3) The SVM technique was used to develop an energy use analysis model and to calculate the sensitivity coefficient of each parameter for coal consumption under different load conditions. The model accuracy of the 300, 350, and 400 MW load condition was lower than that of the 450, 500, 550, and 600 MW load condition. This activity serves as a method to optimize thermal unit operation and minimize energy use.

## Figures and Tables

**Table 1:** Adjustable operating parameters.

Label	Parameter Name	Unit	Label	Parameter Name	Unit
A1	Main steam pressure	MPa	A7	Average smoke temperature	°C
A2	Main steam temperature	°C	A8	Feedwater temperature	°C
A3	Reheat steam temperature	°C	A9	First degree heat desuperheated water	°C
A4	Reheat steam pressure	MPa	A10	Second degree heat desuperheated water	°C
A5	Condenser vacuum	%	A11	Condensate water pressure	MPa
A6	Average smoke oxygen	%	A12	Condensate water temperature	°C

**Table 2:** Reduction in dependence of parameters.

Rank	Parameter	Dependence
1	Average smoke oxygen	0.2537
2	Main steam temperature	0.2509
3	Reheat steam temperature	0.2183
4	Feedwater temperature	0.1774
5	Condenser vacuum	0.1372
6	Main steam pressure	0.1168
7	Average smoke temperature	0.0855

**Table 3:** Clustering results of 400 MW load condition.

Label	Cluster-1	Cluster-2	Cluster-3	Cluster-4	Cluster-5
Main steam temperature (°C)	539.78	538.12	539.15	527.86	538.94
Main steam pressure (MPa)	15.65	15.72	15.65	15.88	15.76
Reheat steam temperature (°C)	536.48	537.50	534.67	523.07	538.67
Average smoke oxygen (%)	5.14	5.54	5.27	5.39	5.15
Average smoke temperature (°C)	113.78	111.90	110.76	115.51	117.38
Condenser vacuum (%)	0.92	0.97	0.95	0.92	0.93
Feedwater temperature (°C)	252.50	250.40	251.38	252.93	251.85
Coal consumption rate (g/(kW·h))	314.53	312.76	315.77	323.53	320.86

**Table 4:** Optimization of reference values under different load conditions.

Load (MW)	300	400	500	600
Main steam temperature (°C)	537.91	538.12	538.52	539.13
Main steam pressure (MPa)	8.35	15.72	16.33	16.55
Reheated steam temperature (°C)	537.71	537.50	538.12	539.78
Average smoke oxygen (%)	6.53	5.54	4.59	3.82
Average smoke temperature (°C)	104.52	111.90	117.23	120.40
Condenser vacuum (%)	0.98	0.97	0.96	0.94
Feedwater temperature (°C)	237.96	250.40	262.18	272.60
Coal consumption rate (g/(kW·h))	320.69	312.76	310.90	307.95

**Table 5:** Parameter sensitivity coefficient of 400 MW load condition.

Parameter	Main Steam Temperature (MPa)	Main Steam Pressure (°C)	Reheated Steam Temperature (°C)	Feedwater Temperature	Average Smoke Oxygen (%)	Average Smoke Temperature (°C)	Condenser Vacuum (%)
Sensitivity valve	0.0245	0.1427	0.0976	0.2202	0.1178	0.0146	0.3827

**Table 6:** Sensitivity analysis model features under different load condition.

Load/MW	Quantity of Training Datasets	Quantity of Test Datasets	Quantity of Support Vector Machines	Average Training Data Error/%	Average Test Data Error Data/%
300	1549	387	1465	0.7606	0.7396
350	1055	264	1038	1.3994	1.2580
400	1067	267	1006	1.0019	0.9232
450	272	68	253	0.3441	0.3875
500	194	49	175	0.2235	0.2390
550	125	31	114	0.2409	0.2412
600	256	64	241	0.4679	0.4477

**Table 7:** Parameter sensitivity coefficient of different load condition.

Load (MW)	300	350	400	450	500	550	600
Main steam pressure (MPa)	0.0028	0.1349	0.0245	0.0856	0.0386	0.0337	0.0288
Main steam temperature (°C)	0.1405	0.1265	0.1427	0.0974	0.1361	0.0862	0.1069
Reheated steam temperature (°C)	0.0026	0.1483	0.0976	0.1571	0.1476	0.1381	0.0973
Feedwater temperature (°C)	0.1698	0.1345	0.2202	0.1749	0.1790	0.2427	0.2859
Average smoke oxygen (%)	0.0551	0.1024	0.1178	0.0355	0.0303	0.0211	0.0190
Average smoke temperature (°C)	0.0929	0.0225	0.0146	0.0326	0.0759	0.1591	0.1739
Condenser vacuum (%)	0.5364	0.2910	0.3827	0.2170	0.4525	0.3933	0.2883
